# Protective role of nano-selenium-enriched *Bifidobacterium longum* in delaying the onset of streptozotocin-induced diabetes

**DOI:** 10.1098/rsos.181156

**Published:** 2018-12-12

**Authors:** Yan Lin, Yongzhe Ren, Yan Zhang, Junjie Zhou, Feng Zhou, Quan Zhao, Genxing Xu, Zichun Hua

**Affiliations:** 1 School of Life Sciences, State Key Laboratory of Pharmaceutical Biotechnology, Nanjing University, Nanjing 210023, Jiangsu, People's Republic of China; 2 School of Nursing, Xinxiang Medical University, Xinxiang 453000, Henan, People's Republic of China; 3 Nanjing Industrial Innovation Center for Pharmaceutical Biotechnology, Nanjing Genrecom Laboratories, Ltd., Nanjing 210031, Jiangsu, People's Republic of China; 4 Changzhou High-Tech Research Institute of Nanjing University, Jiangsu Target Pharma Laboratories Inc., Changzhou 213164, Jiangsu, People's Republic of China; 5 Shenzhen Research Institute, Nanjing University, Shenzhen 518057, Guangdong, People's Republic of China

**Keywords:** *Nano-Se-B. longum*, STZ-induced diabetes, protective effect

## Abstract

*Bifidobacterium longum (B. longum)* could accumulate Selenium (Se) and nano-Se in the form of *Se-B. longum and Nano-Se-B. longum,* respectively*.* In this study, the effect of *Nano-Se-B. longum* in diabetic mice was evaluated. Physiological and metabolic parameters such as blood glucose, body weight, serum insulin level, intraperitoneal glucose tolerance test (IPGTT), food intake, water consumption and urine output were evaluated. The expression of insulin signalling pathway-related proteins was evaluated by western blotting. Haematoxylin and eosin (H&E) was used for histological examination of the liver, pancreas and kidney sections. Creatinine levels in serum (SCr) and blood urea nitrogen (BUN) were measured. *Nano-Se-B. longum* was the best in terms of delaying the onset of diabetes. *Nano-Se-B. longum* decreased blood glucose and body weight compared with those noted for the model group. IPGTT, food intake, water consumption and urine output significantly increased and serum insulin levels significantly decreased in the model group compared with those in all the *Nano-Se-B. longum*-treated mice. Histological results showed that the *Nano-Se-B. longum*-treated mice were better than the model group mice in terms of pathological changes. The expression of insulin signalling pathway-related proteins was upregulated in the *Nano-Se-B. longum*-treated groups. A significant increase in SCr and BUN levels was noted in the model group. This study for the first time reported the dose-dependent preventive effect of *Nano-Se-B. longum* on the onset of diabetes and renal damage. The mechanism may be related to changes in insulin signalling.

## 1. Introduction

*B. longum*, a Gram-positive anaerobic bacterium present in the human gastrointestinal tract, protects the host against viral infections [1,2]. Bifidobacteria are used as probiotics for supporting digestion in many countries [3]. Our previous studies showed that *B. longum* has anti-cancer effects on solid liver cancer [4–9]. To date, numerous studies have demonstrated the benefits of probiotics in managing metabolic disorders including diabetes. Currently, research groups are focusing on this novel concept.

Se, an essential micronutrient for the health of both humans and animals, is actively involved in animal physiology via various selenoproteins [10]. Se supplementation was reported to decrease plasma glucose levels in diabetic rats [11] and humans [12]. Se has also been proven to induce a sustained improvement of glucose homeostasis in diabetic individuals to regulate vital metabolic processes such as glycolysis and gluconeogenesis [13]. The administration of selenium at suitable doses was shown to improve kidney impairments of diabetic kidney disease (DKD) by changing the lipid contents, restoring the ordered structure of the lipids and membrane dynamics [14]. Treatment of diabetic rats with a combination of insulin and Se was effective in controlling blood glucose [15]. However, recent epidemiological studies indicated supranutritional selenium intake and high plasma selenium levels as possible risk factors for the development of type 2 diabetes [16]. Numerous studies have reported the organification of Se through a microorganism fermentation technique for the production of organic Se compounds with higher biological activities and lower toxicity than those of inorganic Se. Organic Se can be absorbed and used rapidly, making it a focus area in recent years [17]. Nano-Se accumulated in *B. longum* existed in the form of selenoproteins and the main component of the organic Se was SeMet. It attracts even more attention thanks to its high bioavailability and lower toxicity. Nano-Se was found to have a hepatoprotective effect, a tumour inhibitory effect, and to improve the immune function of mice [18,19]. These selenoproteins play a preventive role in some degenerative conditions including cancer, inflammatory diseases, neurological diseases, ageing, infertility and infections through specific cellular pathways [20]. Although the mechanisms underlying the anti-diabetic activities of Se are not fully understood, some of the proposed mechanisms include antioxidant protection and stimulation of the immune system. In our previous studies [4,9], we found that *B. longum* could accumulate Se in the form of *Se-B. longum,* affecting tumour growth and immune function in tumour-bearing mice.

Previous studies showed that dietary supplementation with multiple probiotic strains, including *Lactobacillus acidophilus*, *L. casei*, *L. rhamnosus*, *L. bulgaricus*, *B. breve*, *B. longum* and *S. thermophilus*, has been shown to prevent elevations in fasting plasma glucose in diabetic patients [21]. Oral administration of *Bifidobacterium* spp. lowers serum glucose, enhances the expression of proteins involved in the insulin signalling pathway and improves adipokine profile in diabetic mice [22]. A recent study focused on the anti-diabetic effect of *Bifidobacterium* spp. and its molecular mechanism [23]. However, the protective effects of *Nano-Se-B. longum* in a high glucose model have not yet been studied in detail. The effects of *Nano-Se-B. longum* on renal function are also unknown. In this study, wild-type *B. longum (*WT *B. longum), Se-B. longum* and *Nano-Se-B. longum* were used to compare their protective effect on the onset of streptozotocin (STZ)-induced diabetes.

Therefore, we examined whether oral administration of *Nano-Se-B. longum* can delay the onset of STZ-induced diabetes, possibly by affecting the insulin signalling pathway. It was also investigated whether *Nano-Se-B. longum* ameliorates the damage to renal function caused by high glucose levels.

## 2. Material and methods

### 2.1. Nanoparticle formulation and size measurements

Nanoparticles were prepared as described earlier [19]. Briefly, 1 ml of 25 mM sodium selenite (Sangon Biotech Co., Ltd., Shanghai, China), 4 ml of 25 mM reduced glutathione (Sangon Biotech Co., Ltd., Shanghai, China), and 20 mg bovine serum albumin (Sangon Biotech Co., Ltd., Shanghai, China) were mixed. The pH was adjusted to 7.2 with sodium hydroxide, which led to the formation of red nano-Se and oxidized glutathione (GSSG). The red solution was dialysed against double distilled water for 96 h with the water changing every 24 h to separate GSSG from Nano-Se under magnetic stirring. The final solution containing Nano-Se and BSA was subjected to centrifugation at 13 000 r.p.m. for 10 min. The pellet thus recovered was subjected to washing by its re-suspension in deionized water followed by centrifugation at 13 000 r.p.m. for 10 min, to remove possible organic contamination present in the nanoparticles. Finally, the pellet was freeze-dried using a lyophilizer and stored at room temperature. Size measurements were performed using a Zetasizer Nano-ZSE (Malvern Instruments, Malvern, UK) with Zetasizer Software v. 7.12. The results are reported as the average of 40–44 measurements ± s.d.

### 2.2. Preparation of WT *B. longum, Se-B. longum* and *nano-Se-B. longum* strain for administration

*B. longum* NQ-1501 was obtained from the Inner Mongolia Shuangqi Medical Industry Corporation (Inner Mongolia, China) and anaerobically cultured at 37°C in TPY medium. Se enrichment of *B. longum* was performed according to the previously established protocol [8]. Briefly, sodium selenite was purchased from Shanghai LuYuan Fine Chemical Factory, weighed, and dissolved in 200 ml TPY medium at a concentration of 25 µg ml^−1^. Nano red elemental Se was dissolved in 200 ml TPY medium at 5 µg ml^−1^. *B. longum, Se-B. longum* and *Nano-Se-B. longum* were cultivated overnight in TPY medium anaerobically. This overnight culture was diluted 1 : 25 in TPY medium and cultivated at 37°C until the OD_600_ reached about 0.2. The cultured strains were collected and then washed three times with 5% glucose saline by centrifugation at 3500 × *g* for 5 min at 4°C. The collected strains were resuspended in 0.1 ml of 13% milk just prior to use. Live bacteria were prepared daily for administration to each mouse.

### 2.3. Animals

The mice (aged between 4 and 5 weeks (w)) were maintained in a specific pathogen-free animal facility under a 12 h light–dark cycle at an ambient temperature of 21°C. They were provided with water and foods *ad libitum*.

### 2.4. Induction of experimental diabetes

Male mice (C57BL/6) aged 4–5 w were purchased from Nanjing model animal research center of Nanjing University and diabetes was induced with STZ (Merck, Darmstadt, Germany) as previously described [24]. Briefly, after overnight fasting (deprived of food for 12 h and allowed free access to water), diabetes was induced in mice by i.p. injection of STZ dissolved in 0.1 M cold citrate buffer (pH = 4.5) at a dose of 50 mg kg^−1^ body weight for 5 consecutive days. Control mice were injected with citrate buffer alone. Diabetes was confirmed by the determination of fasting blood glucose level on the third-day post-final administration of STZ. Mice with fasting blood glucose levels greater than or equal to 11.1 mM were considered diabetic. Blood glucose levels were monitored every week after diabetes was confirmed using the glucose meter (Sinocare Inc., Changsha, Hunan, China).

### 2.5. Effect of WT *B. longum*, *Se-B. longum* and *nano-Se-B. longum* on glucose level

The effects of WT *B. longum, Se-B. longum* and *Nano-Se-B. longum* on glucose levels of STZ-induced diabetes were determined. The prepared viable organism suspension in 0.1 ml was administered by gavage once a day for 4 w simultaneously. Fifty mice were randomly divided into five groups: Control group—normal; Model group—STZ-induced diabetic mice; WT *B. longum* group, *Se-B. longum* group and *Nano-Se-B. longum* group—STZ-induced diabetic mice treated with 3 × 10^10^ bacteria kg^−1^, respectively. Strain prepared in 0.1 ml of viable microorganism suspension was administered by gavage once a day for 4 w simultaneously. Diabetes was induced in mice by i.p. injection of STZ for 5 consecutive days on the 25th day after strain administered.

### 2.6. Dose-dependent effect of *nano-Se-B. longum*

*Nano-Se-B. longum* prepared in 0.1 ml of viable microorganism suspension was administered by gavage once a day for 4 w simultaneously. Overall, we assessed 60 mice in the six experiments. There were six groups as follows:

Control group: normal;

Model group: STZ-induced diabetic mice;

Low dose group: STZ-induced diabetic mice treated with 7.5 × 10^9^ bacteria kg^−1^ *Nano-Se-B. longum* (treated);

Middle dose group: STZ-induced diabetic mice treated with 1.5 × 10^10^ bacteria kg^−1^ *Nano-Se-B. longum* (treated);

High dose group: STZ-induced diabetic mice treated with 3 × 10^10^ bacteria kg^−1^ *Nano-Se-B. longum* (treated);

Toxicity test group: normal mice treated with 3 × 10^10^ bacteria kg^−1^ *Nano-Se-B. longum*

### 2.7. Physiological assessment and metabolic analysis

The protective effect of *Nano-Se-B. longum* in mice was studied at different doses administered for 4 w. *Nano-Se-B. longum* was administered during the injection of STZ. Blood glucose levels were monitored 3 days to 8 w after the final STZ injection using a glucometer via the caudal vein. Serum insulin levels were determined using Rat/Mouse Insulin ELISA (Millipore Corp, Billerica, MA, USA) at the end of the experiment. Food intake, water consumption and urine output were measured after the mice were placed in metabolic cages overnight. At the eighth week after final STZ injection, eyeball blood was collected and the mice were euthanized. At the end of the experiment, the levels of SCr and BUN were also determined using the assay kit (Nanjing Jiancheng Bioengineering Institute, Nanjing, Jiangsu, China). IPGTT was performed in mice on the seventh week after final STZ injection (*n* = 3). For IPGTT, mice were subjected to an overnight fast followed by an intraperitoneal glucose injection (1.0 g kg^−1^). Blood glucose was measured at 0, 15, 30, 60, and 90 min after the injection.

### 2.8. Western blotting analysis

The liver samples were isolated from all the mice and then snap-frozen in liquid N_2_ for subsequent protein extractions. The collected tissue samples were lysed in ice-cold lysis buffer (20 mM Tris–HCl (pH = 7.5), 150 mM NaCl, 1% Triton-X 100, 1 mM EDTA) and a protein inhibitor cocktail for 30 min. The supernatant was boiled with Laemmli sample buffer for SDS-PAGE. The following antibodies were used: anti-IRS1, anti-phospho-IRS1 (pIRS1), anti-GSK-3*β*, anti-phospho-GSK-3*β* (pGSK-3*β*), anti-AKT and anti-phospho-AKT (pAKT) (Thr308) (Cell Signaling Technology, Beverly, MA); anti-β-actin monoclonal antibody, anti-α-tubulin and anti-GAPDH were purchased from Santa Cruz Biotechnology Inc. (Santa Cruz, Delaware, USA). Goat anti-rabbit IgG and goat anti-mouse IgG were from Jackson ImmunoResearch Europe Ltd. The band densities were quantified by using Image J program.

### 2.9. Histological analysis

To observe the morphological changes of the liver, pancreas and kidney, H&E staining was carried out as described before [25]. In brief, the liver, pancreas and kidney tissues were fixed in 4% paraformaldehyde for 16–24 h and transferred to ethanol. Then, the samples were embedded in paraffin and sectioned at 5 µm, followed by H&E staining.

### 2.10. Statistical analysis

Data are presented as means ± SEM. The difference between two groups was analysed by a two-tailed Student's *t*-test using Prism software (GraphPad, San Diego, CA). Values were considered statistically significant at *p* < 0.05.

## 3. Results

### 3.1. Effects of WT *B. longum, Se-B. longum* and *nano-Se-B. longum* on glucose levels of STZ-induced diabetes

**Figure 1.**
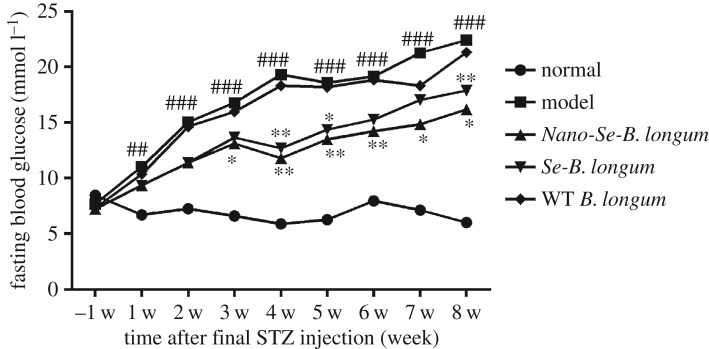
The effects of WT *B. longum, Se-B. longum and Nano-Se-B. longum* on glucose. Blood glucose measurement of the mice during the fasting course at 1 w before STZ injection and at 1–8 w after final STZ injection. Data are presented as means ± s.e.m. (*n* = 10 per group). Statistical significance was assessed by two-tailed Student's *t*-test. (^##^*p* < 0.01, ^###^*p* < 0.001 compared with normal group; *p < 0.05, **p < 0.01 compared with the model group).

*Nano-Se-B. longum* exhibited the best effect on fasting blood glucose levels (figure 1). Thus, *Nano-Se-B. longum* was chosen for further studies.

### 3.2. Effects of *nano-Se-B. longum* on physiological and metabolic parameters

**Figure 2.**
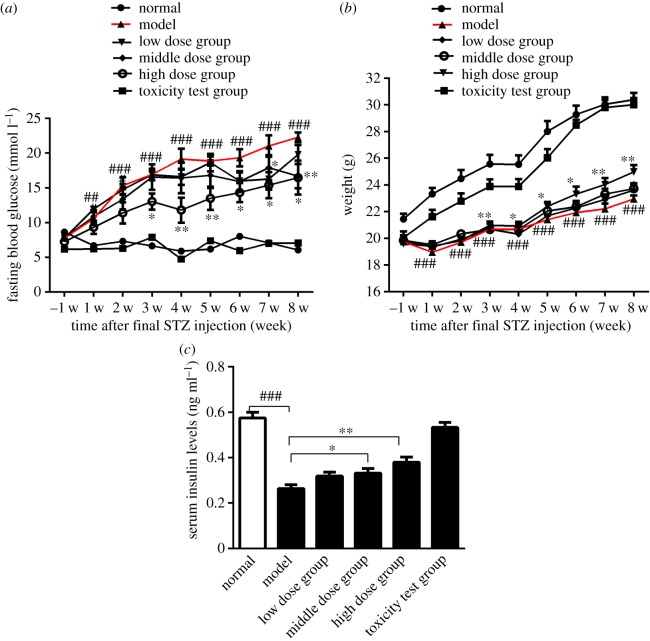
The measurement of the fasting blood glucose and weight. (*a*) The test of blood glucose during the fasting course at 1 w before STZ injection and at 1–8 w after final STZ injection. (*n* = 9 per group). (*b*) The test of the fasting weight at 1 w before STZ injection and at 1–8 after final STZ injection (*n* = 9 per group). (*c*) Fasted serum insulin levels in mice at 8 w after final STZ injection (*n* = 6 per group). Data are presented as means ± s.e.m. Statistical significance was assessed by two-tailed Student's *t*-test. (^##^*p* < 0.01, ^###^*p* < 0.001 compared with normal group; *p < 0.05, **p < 0.01 compared with the model group).

**Figure 3.**
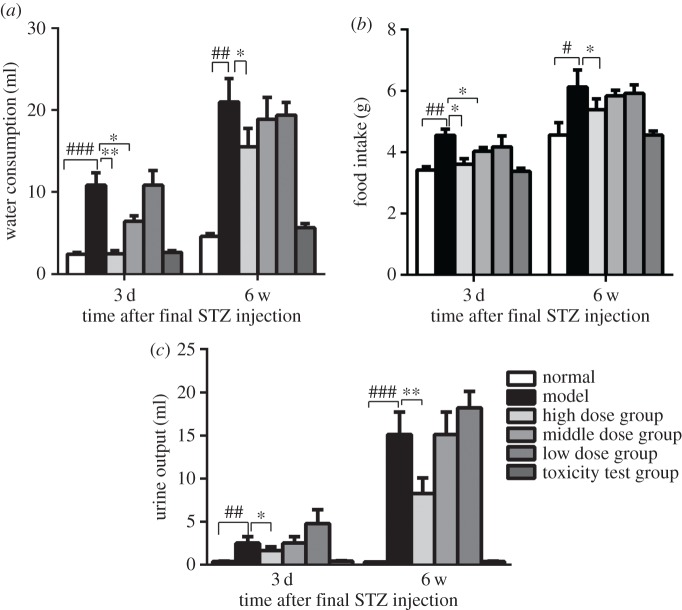
Effect of *Nano-Se-B. longum* on metabolic condition. Metabolic studies using metabolic cages show food intake (*a*), water consumption (*b*) and urine output (*c*) in all the mice on day 3 and at week 6 after final STZ injection. Data are presented as means s.e.m. (*n* = 10 per group). Statistical significance was assessed by two-tailed Student's *t*-test. (^##^*p* < 0.01, ^###^*p* < 0.001 compared with normal group; *p < 0.05, **p < 0.01 compared with the model group).

**Figure 4.**
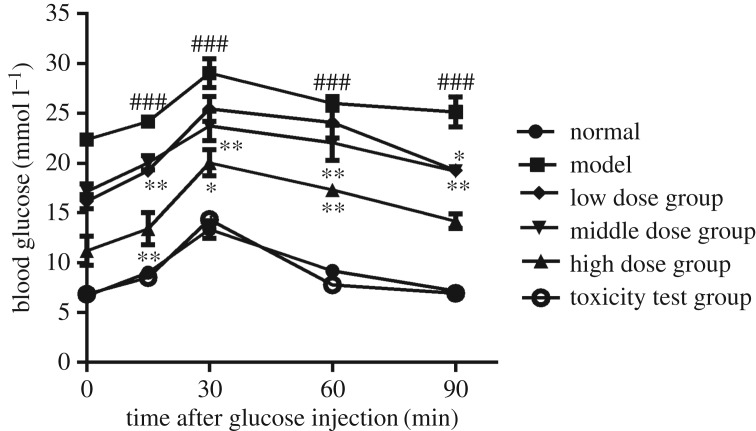
The effect of *Nano-Se-B. longum* on glucose homeostasis. IPGTT of all the mice was performed in mice on the seventh week after final STZ injection. Mice were injected intraperitoneally with 1.0 g kg^−1^ glucose, and blood glucose levels were monitored at the intervals indicated. Data were shown as mean ± s.e.m. (*n* = 3 per group). Statistical significance was assessed by two-tailed Student's *t*-test. (^###^*p* < 0.001, compared with normal group; *p < 0.05, **p < 0.01, compared with the model group).

Blood glucose testing is the gold standard for the subclinical diagnosis of diabetes. *Nano-Se-B. longum*-treated mice exhibited notably lower fasting blood glucose levels (figure 2*a*) and higher body weight (figure 2*b*) than model mice. Because glucose homeostasis is mainly regulated by insulin, we also detected its serum concentration (*n* = 6). Fasting insulin levels were higher in *Nano-Se-B. longum*-treated mice (figure 2*c*) than in model mice. Twenty-four-hour food intake, water intake and urine volume were measured (*n* = 10) and found to be decreased with an increase in the dosage of *Nano-Se-B. longum* (figure 3). In the IPGTT assay (*n* = 3), the glucose levels decreased significantly in model group mice (figure 4), indicating an improved glucose clearance after *Nano-Se-B. longum* intervention in a dose-dependent manner.

### 3.3. Effects of *nano-Se-B. longum* on morphological changes in the liver and pancreas

**Figure 5.**
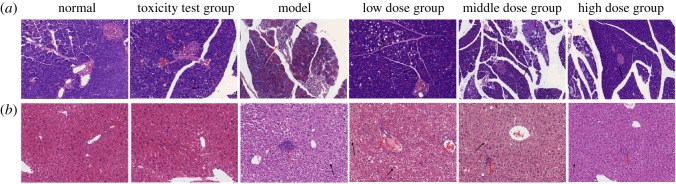
Effect of *Nano-Se-B. longum* on the pancreas and liver morphological changes (H&E stain; 20 × 10). Histopathological observations made on the pancreas (*a*) and liver (*b*) of experimental groups of mice and the photomicrographs presented are the representatives of the eight mice used in each group. Representative images are shown, at a magnification of 200.

Histological analysis of the liver and pancreas by H&E staining showed a notable difference between *Nano-Se-B. longum*-treated and control mice. As shown in figure 5*a*, there were no obviously harmful changes in the control mice and toxicity test group mice. A small amount of fat vacuoles was observed in part of the pancreatic section in the model, low, middle, and high dose groups (black arrow). Small amounts of inflammatory cells were only visible in the tissue in the model group (red arrow). With the increase in the dosage of *Nano-Se-B. longum,* the degree of lesion decreased gradually. As shown in figure 5*b*, no obviously harmful changes in the control mice and toxicity test group were noted. The hepatic cells were edematous and the cytoplasm was loose in the tissue (black arrow) in the STZ-treated groups, while the degree of lesion decreased gradually with an increase in the dosage of *Nano-Se-B. longum*. Small amounts of inflammatory cells were visible in the tissue (red arrow) in the model group and the degree of infiltrated inflammatory cells decreased with an increase in the dosage of *Nano-Se-B. longum* (red arrow). Overall, the progression of liver and pancreas pathological damage was slowed after *Nano-Se-B. longum* treatment.

### 3.4. *Nano-Se-B. longum* improved liver insulin signalling sensitivity

**Figure 6.**
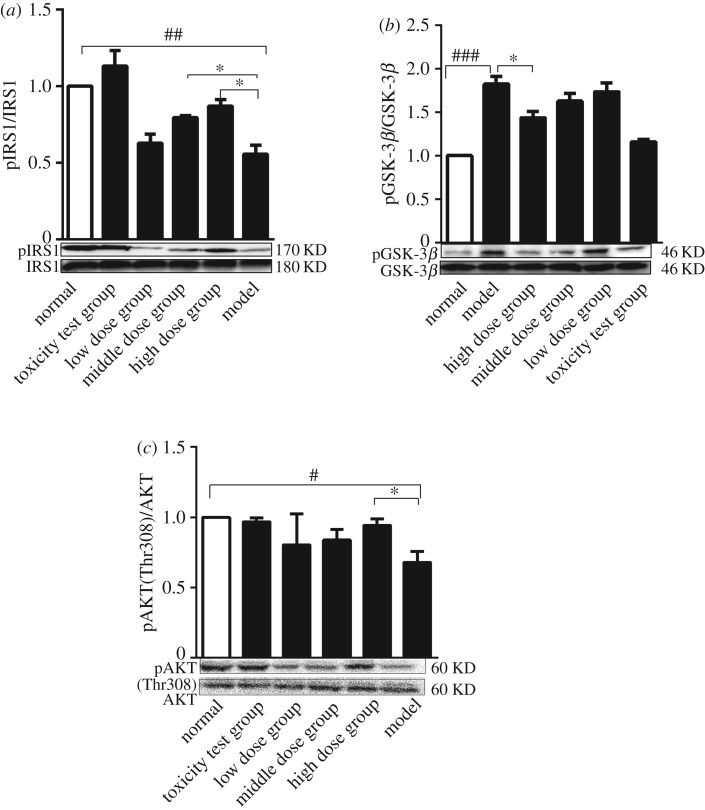
The analysis of the insulin signalling pathway. Liver pIRS1 (*a*), pGSK-3*β* (*b*) and pAKT (*c*) protein levels were measured by western blot analysis, which were normalized to IRS1, GSK-3*β* and AKT, respectively. Data were shown as mean s.e.m. (three mice per group). Statistical significance was assessed by two-tailed Student's *t*-test. (^#^*p* < 0.05, ^##^*p* < 0.01, ^###^*p* < 0.001, compared with normal group; *p < 0.05 compared with the model group).

To investigate the molecular mechanisms underlying hypoglycaemia, we studied the insulin signalling pathway, which plays a critical role in glucose homeostasis. The mice were assessed for the presence of pIRS1, pGSK-3*β* and pAkt (Thr308). As shown in figure 6*a,c,* the expression of pIRS-1 and pAkt increased significantly in the liver from the treatment group compared with that in the control mice. pGSK-3*β* levels decreased markedly in the trial group compared with that in control mice (figure 6*b*). The expression of insulin signalling pathway proteins was upregulated, which showed that *Nano-Se-B. longum* improved liver insulin signalling sensitivity.

### 3.5. Protective role on renal function

**Figure 7.**
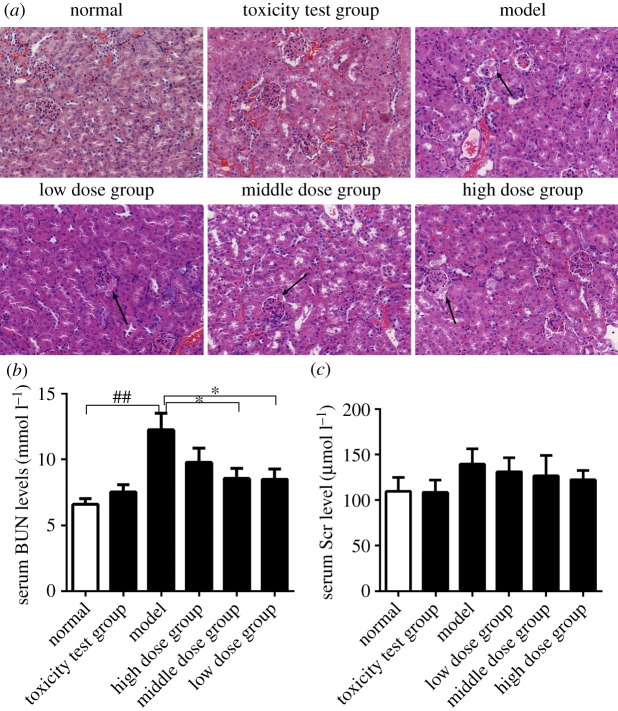
The protective role in the renal function. (*a*) Micrographs of renal sections stained with H&E. (*b*) The levels of BUN in serum (*n* = 6 per group). (*c*) The levels of SCr in serum. Data were shown as mean ± s.e.m. (*n* = 6 per group). Statistical significance was assessed by two-tailed Student's *t*-test. (^##^*p* < 0.01 compared with normal group; *p < 0.05 compared with the model group).

The influence of *Nano-Se-B. longum* on the kidney is attributable to its effects on the glomeruli. With the increase in *Nano-Se-B. longum* dosage, mesentery cell hyperplasia and glomerulus atrophy decreased gradually (black arrow) (figure 7*a*). *Nano-Se-B. longum* markedly decreased the levels of BUN and SCr in serum in STZ-induced mice compared to control mice (figure 7*b*,*c*). These data suggest that *Nano-Se-B. longum* may improve the renal function damaged by diabetes.

## 4. Discussion

In this study, the effects of WT *B. longum, Se-B. longum* and *Nano-Se-B. longum* on glucose were measured. The results showed that *Nano-Se-B. longum* was the best with respect to the protective effect on high blood glucose. Normal mice were treated with the maximum dose of *Nano-Se-B. longum* and no significant difference was observed compared to normal mice. Sudin Bhattacharya research group had synthesized and characterized Nano-Se and found its chemoprotective (CP) activity against CP-induced hepatotoxicity, pulmonary and genotoxicity in normal Swiss albino mice [19,26] and its anti-tumour efficacy in the tumour-bearing Swiss albino mice [18]. The anti-genotoxic effect of Nano-Se might be due to its antioxidant and cytoprotective activity. Now, *Nano-Se-B. longum* showed its safety and protective effect in STZ-induced diabetes. We have expanded the functions of Nano-Se, providing further understanding and insight. Some studies found that the restorative effect of selenium on diabetes is predominantly related to the antioxidant and insulin-like properties of selenium [14]. However, further studies are required to investigate the precise mechanisms involved in the protective effect of *Nano-Se-B. longum* against diabetes.

The insulin signalling pathway controls glucose transport in liver cells. Insulin binds to insulin receptors on the surfaces of target cells. This binding activates insulin receptor beta (IR-β), and then activates IRS1, thereby recruiting phosphatidylinositol 3-kinase (PI3 K) to this location. An important target of PI3 K in liver cells is Akt/PKB, which has a key function in glucose uptake [27]. Previous studies have shown that pIRS1 and pAkt upregulation may have improved glucose uptake by the reduced plasma glucose levels [28]. Oral administration of *Nano-Se-B. longum* may give rise to elevated plasma selenium levels by enhanced hepatic secretion of selenoproteins, which may enhance insulin-induced signal transduction [16]. Therefore, we assessed the effects of *Nano-Se-B. longum* administration on insulin signalling pathways. In our study, *Nano-Se-B. longum* increased the levels of pIRS1 and pAkt proteins and decreased pGSK-3*β* in diabetic mice. We can reasonably speculate that an increase in the selenoproteins induced by *Nano-Se-B. longum* treatment enhanced insulin sensitivity by promoting the insulin signalling pathway.

Diabetes mellitus can cause serious health problems including macrovascular and microvascular complications [29]. One of these is injuries to the kidney tissue that result in renal dysfunction [30]. Eight weeks after STZ diabetes induction, some indexes of renal damage such as an increase in BUN were noted [31]. There is a large amount of evidence to support the recovery effects of selenium, at suitable doses, on the cell membrane of diabetic kidneys. The beneficial effect of selenium on renal lesions can be explained with its insulin-like effect [32]. Recently, Feride Severcan *et al.* [14] also showed the efficiency of a low dose (1 µmol kg^−1^) of selenium administration in the prevention of diabetes-related complications in kidneys. We also investigated the renoprotective effect of *Nano-Se-B. longum* in STZ-induced mice. *Nano-Se-B. longum* can decrease renal dysfunction by lowering BUN and SCr. Our experiments in *Nano-Se-B. longum*-treated and STZ-induced diabetes mice revealed that *Nano-Se-B. longum* exerts a protective role in delaying the onset of STZ-induced diabetes as well as renal function. However, further studies are required to investigate the precise mechanisms involved in the renoprotective effect.

Our findings may facilitate the understanding of the novel effects of *Nano-Se-B. longum* and suggest a newly recognized benefit of *Nano-Se-B. longum* in diabetic mice. This may provide a novel, feasible, economic protection approach for diabetes, thus deserving further investigation and development.

## 5. Conclusion

In this study, we demonstrated that oral administration of *Nano-Se-B. longum* can delay the onset of STZ-induced diabetes, possibly via its effect on the insulin signalling pathway. It was also investigated that *Nano-Se-B. longum* ameliorates the damage of renal function caused by high glucose levels. Our findings may facilitate the understanding of the novel effects of *Nano-Se-B. longum* and suggest a newly recognized benefit of *Nano-Se-B. longum* in diabetic mice.

## Data accessibility

Our data are deposited at Dryad Digital Repository: (http://dx.doi.org/10.5061/dryad.v4g40ms) [33].
